# Metabolomics reveals effects of maternal smoking on endogenous metabolites from lipid metabolism in cord blood of newborns

**DOI:** 10.1007/s11306-016-0983-z

**Published:** 2016-03-08

**Authors:** Ulrike E. Rolle-Kampczyk, Jan Krumsiek, Wolfgang Otto, Stefan W. Röder, Tibor Kohajda, Michael Borte, Fabian Theis, Irina Lehmann, M. von Bergen

**Affiliations:** Department of Metabolomics, Helmholtz Centre for Environmental Research – UFZ Leipzig, Leipzig, Germany; Institute of Computational Biology, Helmholtz Centre Munich & Mathematics, Oberschleißheim, Germany; Department of Proteomics, Helmholtz Centre for Environmental Research – UFZ Leipzig, Leipzig, Germany; Core Facility Studies, Helmholtz Centre for Environmental Research – UFZ Leipzig, Leipzig, Germany; Hospital “St. Georg” GmbH Leipzig, Academic Teaching Hospital of the University of Leipzig, Delitzscher Straße 141, 04129 Leipzig, Germany; Department of Environmental Immunology, Helmholtz Centre for Environmental Research – UFZ Leipzig, Leipzig, Germany; TU Munich, Munich, Germany; Faculty of Biosciences, Pharmacy and Psychology, Institute of Biochemistry, Universität Leipzig, Leipzig, Germany; Department of Chemistry and Bioscience, Center for Microbial Communities, Aalborg University, Aalborg, Denmark

**Keywords:** Smoking, Pollutant derivatives, Serum metabolom, Cord blood

## Abstract

**Introduction:**

A general detrimental effect of smoking during pregnancy on the health of newborn children is well-documented, but the detailed mechanisms remain elusive.

**Objectives:**

Beside the specific influence of environmental tobacco smoke derived toxicants on developmental regulation the impact on the metabolism of newborn children is of particular interest, first as a general marker of foetal development and second due to its potential predictive value for the later occurrence of metabolic diseases.

**Methods:**

Tobacco smoke exposure information from a questionnaire was confirmed by measuring the smoking related metabolites S-Phenyl mercapturic acid, S-Benzyl mercapturic acid and cotinine in maternal urine by LC–MS/MS. The impact of smoking on maternal endogenous serum metabolome and children’s cord blood metabolome was assessed in a targeted analysis of 163 metabolites by an LC–MS/MS based assay. The anti-oxidative status of maternal serum samples was analysed by chemoluminiscence based method.

**Results:**

Here we present for the first time results of a metabolomic assessment of the cordblood of 40 children and their mothers. Several analytes from the group of phosphatidylcholines, namely PCaaC28:1, PCaaC32:3, PCaeC30:1, PCaeC32:2, PCaeC40:1, and sphingomyelin SM C26:0, differed significantly in mothers and children’s sera depending on smoking status. In serum of smoking mothers the antioxidative capacity of water soluble compounds was not significantly changed while there was a significant decrease in the lipid fraction.

**Conclusion:**

Our data give evidence that smoking during pregnancy alters both the maternal and children’s metabolome. Whether the different pattern found in adults compared to newborn children could be related to different disease outcomes should be in the focus of future studies.

**Electronic supplementary material:**

The online version of this article (doi:10.1007/s11306-016-0983-z) contains supplementary material, which is available to authorized users.

## Introduction

With about 250 million smoking women worldwide naturally also a huge number of newborn children are affected by tobacco smoke. Many components of tobacco smoke have been shown to cross the placenta (Jordanov 1990) and for nicotine even a concentrating effect in the foetal blood was found. Prenatal tobacco smoke exposure is regarded as the most frequent avoidable cause of adverse health effects on the foetus such as preterm birth and intrauterine growth retardation (Ward et al. 2007; Windham et al. 2000). Furthermore a correlation between maternal smoking and the risk for developing obesity in the offspring has been reported in epidemiological studies (Oken et al. 2005). Since the mechanistic link between prenatal tobacco smoke exposure and overweight development of the child still remains unclear profiling the metabolome of the affected children seems to be a reasonable strategy.

The effect of smoking on serum metabolites in adults has been described by Wang-Sattler et al. (2008a) using a targeted approach that detected and quantified 198 endogenous metabolites. In that study the groups of current smokers were clearly discriminated from non-smokers, whereas the former smokers were only partially separated by multivariate analysis. The strongest metabolite concentration differences were found for phosphatidylcholines, which belong to fatty acid metabolism. Within this larger group of phosphatidylcholines especially the balance between the acyl–acyl and the acyl-alkyl forms was affected.

The assessment of the metabolomic changes in a limited volume of fetal blood became possible with the advent of highly sensitive mass spectrometry devices. To the best of our knowledge, this technique has so far only once been applied in one newborn study focussing on metabolomic profiles of children with intrauterine growth restriction (Favretto et al. 2012). Favretto et al. used an untargeted LC–MS approach and detected many peaks that provided hints for clear separation between controls and those newborn children with restricted growth based on principal component analysis. Among others, the essential amino acids phenylalanine, tryptophan, and methionine were identified to be differentially abundant in children with restricted intrauterine growth vs controls. In further studies the metabolome profile occurring upon intrauterine growth restriction has been analysed in pigs (Nissen et al. 2011; Rudashevskaya et al. 2012) by applying NMR. In these studies the difference between normal foetal weight and low foetal weight were pinpointed to a broad range of alterations from amino acids to various molecules in lipid metabolism.

The potential of screening approaches lies in their inherent potential to discover alterations in so far unexpected metabolites. On the other hand, in untargeted approaches many peaks are hard to identify why the full potential in terms of coverage is seldom fully tapped. For assessing the metabolome of the maternal and foetal blood of non- and smoking mothers we decided to use a highly similar and thereby comparable targeted approach as applied by Wang-Sattler et al. (2008a) for the characterization of nicotine dependent metabolome changes in an adults. Since a highly similar measurement was used in both studies the data will be well comparable.

The samples were collected in the framework of a prospective cohort study designed for the investigation of prenatal environmental exposure on the risk of the child for developing atopic diseases (Herberth et al. 2011). In this study the parental smoking habit is assessed by questionnaire. In addition, tobacco-smoke related metabolites (S-phenyl mercapturic acids as specific metabolite for benzene and S-benzyl mercapturic acid as specific metabolite of toluene) were analysed in maternal urine at the 34th week of gestation. Benzene and toluene are volatile components of tobacco smoke and smoking is regarded as the main source of benzene in indoor air. Urinary mercapturic acids were described as biomarkers for exposure in the early 1990s (De Rooij et al. 1998; Haufroid and Lison 2005) and the validity of them has been demonstrated in numerous studies (Inoue et al. 2004; Takahashi et al. 1994), (Barbieri et al. 2008; Rolle-Kampczyk et al. 2002). As environmental tobacco smoke (ETS) is known to cause oxidative stress, we also tested for the antioxidative capacity in the serum of the mothers.

Beyond the measurement there is an urgent need for developing evaluation tools of metabolome data. The classical way of calculating significant changes between single metabolites in different conditions and presentation of principal component analysis plots provides the basic analysis but does not tap the full potential of interpreting high dimensional metabolome data. For further analysis first the data-driven correlation analysis is important to identify co-regulation of metabolites. However, this has to be corrected by biochemical connectivity in order to generate pathway dependent information which is much more robust than single biomarker based monitoring of cellular reactions Krumsiek et al. (2011a; Wang-Sattler et al. 2008a).

For the first time we are describing here the cord blood metabolome and the impact of prenatal tobacco smoke exposure on the foetal metabolism. Obtained results may help to explain the link between prenatal exposure to smoke contaminants and the metabolomic programming of the children.

The following hypotheses can be formulated:

1. Mothers and newborn children show a similiar metabolom regulation.
2. Metabolic changes are detectable for mothers and newborns depending on the mother´s smoking behavior.
3. It is possible to show different concentrations of the smoking related metabolites S-phenyl and S-benzyl mercapturic acid and cotinine.
4. The antioxidative status is smoke burdened associated.

## Material and methods

### Study design

 The LINA study (Lifestyle and Environmental Factors and their Influence on Newborn Allergy risk) was designed to investigate the prenatal influence of life style and environmental factors on the maturation of the immune system and the resulting risk in developing atopic diseases in infancy. 629 mother–child pairs (622 mothers; 7 twin pairs) were recruited in Leipzig, Germany in the Children’s Hospital “St. Georg” between May 2006 and December 2008 (Herberth et al. 2011). Information on the mother`s and father`s allergy status, living conditions, and environmental exposure was gathered by way of questionnaires. The smoking mothers were slightly younger than the smoke-free mothers (27.4 years vs 30.4 years). The smoking behaviour was included in the analysis as a confounding factor. Parental asthma was included as a confounder with other confounding factors. We do not have information on parental diabetes. One inclusion criterion for the study was: no persistent disorders. Therefore medication on a regular basis (i.e. during pregnancy) is uncommon in our cohort. We removed all participants who could not fulfill inclusion criteria.

We defined exclusion criteria, namely the diagnosis of asthma and diabetes, and furthermore we excluded these persons who showed drug abuse, as detected in a recent study (Hoeke et al. 2015).

During a clinical visit at the 34th week of pregnancy venous blood and urine samples were collected from the mother. After birth, cord blood was obtained by venous puncture. Participation in the study was voluntary. Written informed consent was obtained from the parents. The study was approved by the Ethics Committee of the University of Leipzig (reference number 046-2006).

For this study a subsample of 35 mother child pairs (17 smoking and 18 non-smoking mothers and their children and ever 2 smoking and non-smoking mother and one additional smoke burdend child) was randomly selected. For differentiation in non-smoker and smoker group we used information from questionnaires as well as the urine cotinine level (above 30 µg cotinine/g creatinine for the smoke burdened group). The here studied subgroup of children contains 28 boys (70 %) and 12 girls (30 %), from which 31 (77 %) were born spontaneous. The average birth weight was 3434 g (range 2610–4165 g). Only full term neonates, born after 37th week of gestation with a birth weight above 2.500 g were included into this analysis.

### Material sampling

Urine was personal sampled as morning urine by mothers self on the day of house visits. After that the samples were transferred to the lab, than sterile filtered deep frozen (−80 °C) up to analysis.

Mother´s blood were collected during consultation hour (34th week of gestation). Cord blood samples were collected during birth in maternity room and handled as described elsewhere (Hinz et al. 2012). In brief, serum was prepared immediately and samples were transferred to and stored in −80 °C deep freezer up to analysis. An interruption of freezing can be excluded by authors.

### Determination of smoking-related exogenous metabolites and the oxidative status of serum samples

The measurement of tobacco smoke metabolites in maternal urine was performed as described elsewhere (Rolle-Kampczyk et al. 2006). The anti-oxidative status of maternal serum samples was analysed by Photochem^R^ (Analytic Jena, Germany). After calibration using standards provided by the supplier 10 µl of serum were mixed with 2290 µl working solution 1. Afterwards 200 µl reaction buffer and 25 µl stock solution was added. The total mixture was then placed into the sampling device and immediately measured. The instrument uses the calibration for internal calculation of standardized values of antioxidant capacity.

### Assessment of endogenous metabolites

The metabolome analyses were carried out using the Absolute*IDQ*^®^ p150 Kit (Biocrates Life Science AG, Innsbruck, Austria) as described elsewhere (Oberbach et al. 2012). In brief, 10 µL of serum were mixed with internal standard and dried under nitrogen. Subsequently the metabolites were derivatized with phenylisothiocyanate (PITC) 5 % for 20 min at room temperature and dried for 30 min under nitrogen flow. For extraction first 300 µL of extraction solvent (5 mM ammonium acetate in methanol) were added and incubated with shaking at 450 rpm (Thermomixer comfort Eppendorf, Hamburg, Germany) for 30 min at room temperature followed by filtration by centrifugation (Sigma 2–16 k, Taufkirchen, Germany) for 2 min at 500 × g. From the filtrate 100 µL were mixed with 500 µl of MS running buffer and 20 µL were subjected for flow injection analysis-MS/MS measurements (FIA-MS/MS). MS measurements were performed on a QTRAP mass spectrometer applying electrospray ionization (ESI) (ABI Sciex API5500Q-TRAP). Identification and quantification were achieved by multi reaction monitoring (MRM) standardized by applying spiked-in isotopically labelled standards in positive and negative mode, respectively. For calibration a calibrator mix consisting of 7 different concentrations was used. Quality controls were included for 3 different concentration levels. For FIA an isocratic method was used (100 % organic running solvent) with varying flow conditions (0 min, 30 µL/min; 1.6 min 30 µL/min; 2.4 min, 200 µL/min; 2.8 min, 200 µL/min; 3 min 30 µL/min), and the MS settings were as follows: Scan time 0.5 s, IS voltage for positive mode 5500 V, for negative mode −45,000 V, nitrogen as collision gas medium, source temperature 200 °C. The integrated MetIDQ software (Biocrates, Innsbruck, Austria) streamlines data analysis by automated calculation of metabolite concentrations providing quality measures and quantification.

### Data evaluation and statistical analysis

Statistical analyses were performed using the R platform version 2.14.1 (http://www.r-project.org). Besides the basic packages in R, we used Car, Coin, Corrgram, Gdata and Psych. Non parametric Wilcoxon tests were performed for each analyte to check for significant differences between the two considered groups. Pearson’s correlation coefficients were determined to analyse pair wise correlation of the metabolites. For PCA and clustering, the concentrations of the samples have been scaled to mean = 0 and standard deviation = 1. The PCA was calculated via the princomp function in R while clustering is performed by the hclust function based on the Euclidean distances of the concentrations.

### Differential correlation analysis

Before differential correlation analysis, a quality control procedure was applied in order to avoid false negatives and spurious correlations. First, variables with more than 50 % of values below the detection limit were excluded. Second, we removed outlier samples that deviate more than 3 standard deviations from the respective mean for at least one metabolite. After these preprocessing steps the data matrix contained 60 metabolites and 75 samples. Pairwise Pearson correlations for the four groups (mother/smoker, mother/non-smoker, cord blood/smoker, cord blood/non-smoker) were calculated separately and differential correlation analysis between groups was performed. The significance of correlation differences between two groups *A* and *B* for two given metabolites $i$ and *j* was assessed by the following test statistic:

$$T_{ij}^{AB} = \frac{{z\left( {\rho_{ij}^{A} } \right) - z\left( {\rho_{ij}^{B} } \right)}}{{\sqrt {\frac{1}{{n_{A} - 3}} + \frac{1}{{n_{B} - 3}}} }},$$

where *ρ*_*ij*_^*A*^ and *ρ*_*ij*_^*B*^ represent the Pearson correlations between i and j in the respective groups, *n*_*A*_ and *n*_*B*_ are the sample sizes of both groups, and *z*( · ) represents the Fisher transformation $z\left( x \right)\text{ := } \frac{1}{2}\ln \left( {\frac{1 + x}{1 - x}} \right)$. *T*_*ij*_^*AB*^ is approximately standard normally distributed. A two-sided *p* value can be derived from the respective quantile of the standard normal distribution.

## Results

In order to analyse clearly defined groups the mothers were selected according to their questionnaire answers on their smoking habit (Herberth et al. 2011; Hinz et al. 2010). In addition to questionnaire information, the current maternal tobacco smoke exposure status was validated by chemical assessment of the smoking-derived metabolites S-phenyl mercapturic acids, S-benzyl mercapturic acid, and cotinine in the urine collected at 34th week of gestation.

**Fig. 1:**
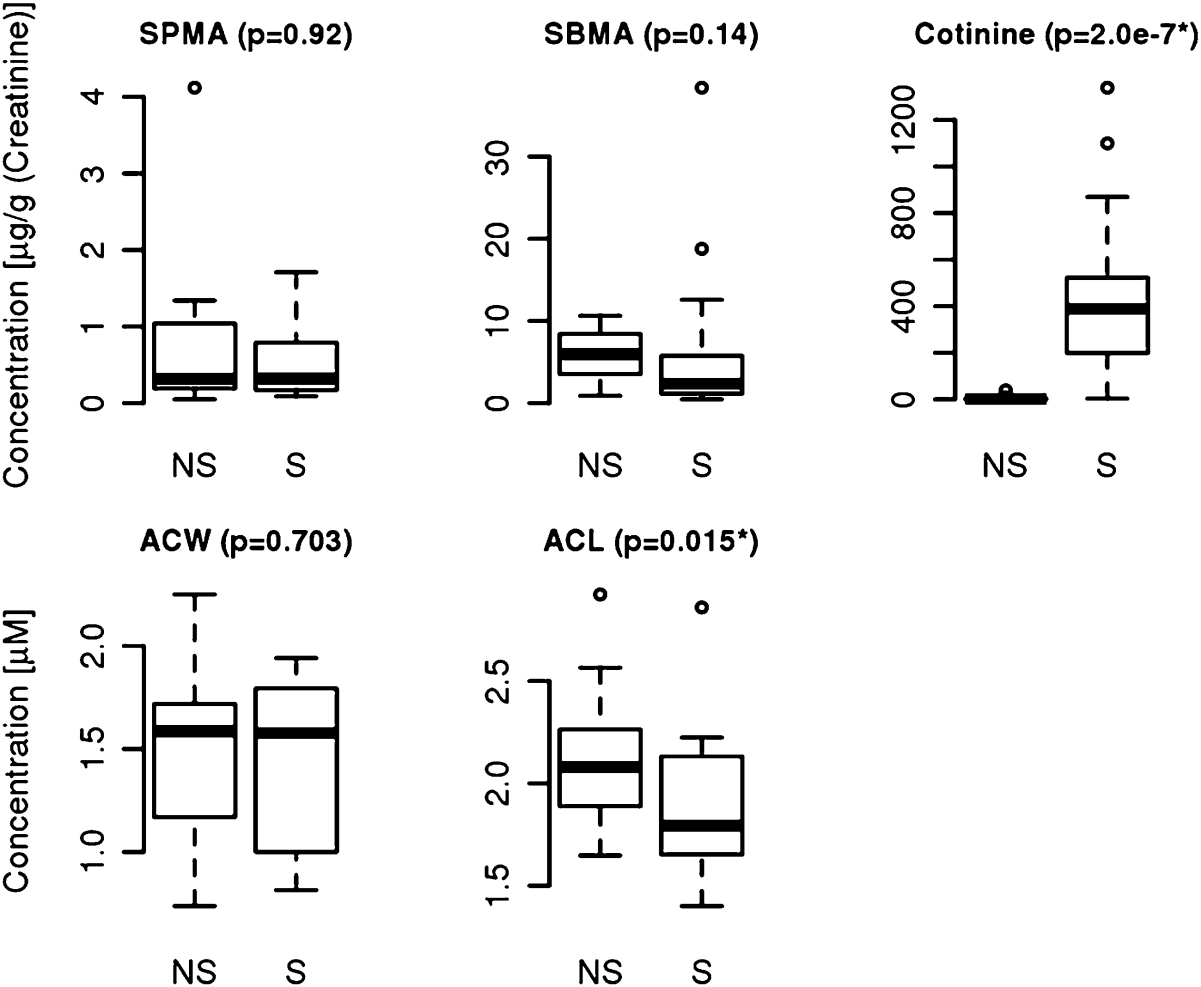
Exogenous metabolites (*S*-phenyl mercapturic acid (SPMA), *S*-benzyl mercapturic acid (SBMA) and Cotinine) in urine of mothers at 34th week of gestation for non-smoker (NS) and smoker (S) and antioxidative status of water soluble (ACW) and lipid soluble compounds (ACL) in maternal blood for non-smoker (NS) and smoker (S)

The urine concentrations of the benzene and toluene derivates S-phenyl mercapturic acids (SPMA: 0.71 ± 0.92 vs 0.58 ± 0.53 µg/g creatinine) and S-benzyl mercapturic acid (SBMA: 6.08 ± 2.8 vs 6.60 ± 9.31 µg/g creatinine) did not differ significantly between smokers and non-smokers (Fig. 1). Cotinine is the utmost important nicotine metabolite Benowitz et al. (2009) and has been proven to be a suitable marker to differentiate between smoke burdened and unburdened persons (Benowitz 1996). Here we found clear differences between non-smokers (2.12 ± 2.07 µg/g creatinine) and smokers (411.37 ± 351.41 µg/g creatinine) that are highly significant (p = 2 × 10^−7^). Also the correlation between the questionnaire items on smoking/ETS exposure and urine cotinine levels was positive (R = 0.65, spearman’ rank correlation).

Another reported consequence of smoking is oxidative stress (Zalata et al. 2007). To further confirm the effects of smoking we analysed the antioxidant capacity in the serum. The maternal blood was analysed by a chemoluminiscence based method making use of the fast photochemical excitation of radical formation combined with sensitive luminometric detection. The water soluble antioxidant capacity (ACW) measurements summarize the effects of ascorbinic acid, uric acid and bilirubine. The lipid soluble capacity (ACL) represents the aggregated antioxidative capacity of ß-carotine, Q10-Enzyme and tocopherol. In this study we found no significant difference in the water soluble fraction between smoking and non-smoking mothers (1.49 ± 0.43 mmol/mL non-smoker group vs 1.3 ± 0.42 mmol/mL smoker group). In contrast, the antioxidative potential in the lipid soluble fraction was higher in the serum of non-smoking mothers (2.12 ± 0.32 mmol/mL) compared to the smoking group (1.89 ± 0.26 mmol/mL, p = 0.015, Fig. 1).

**Fig. 2:**
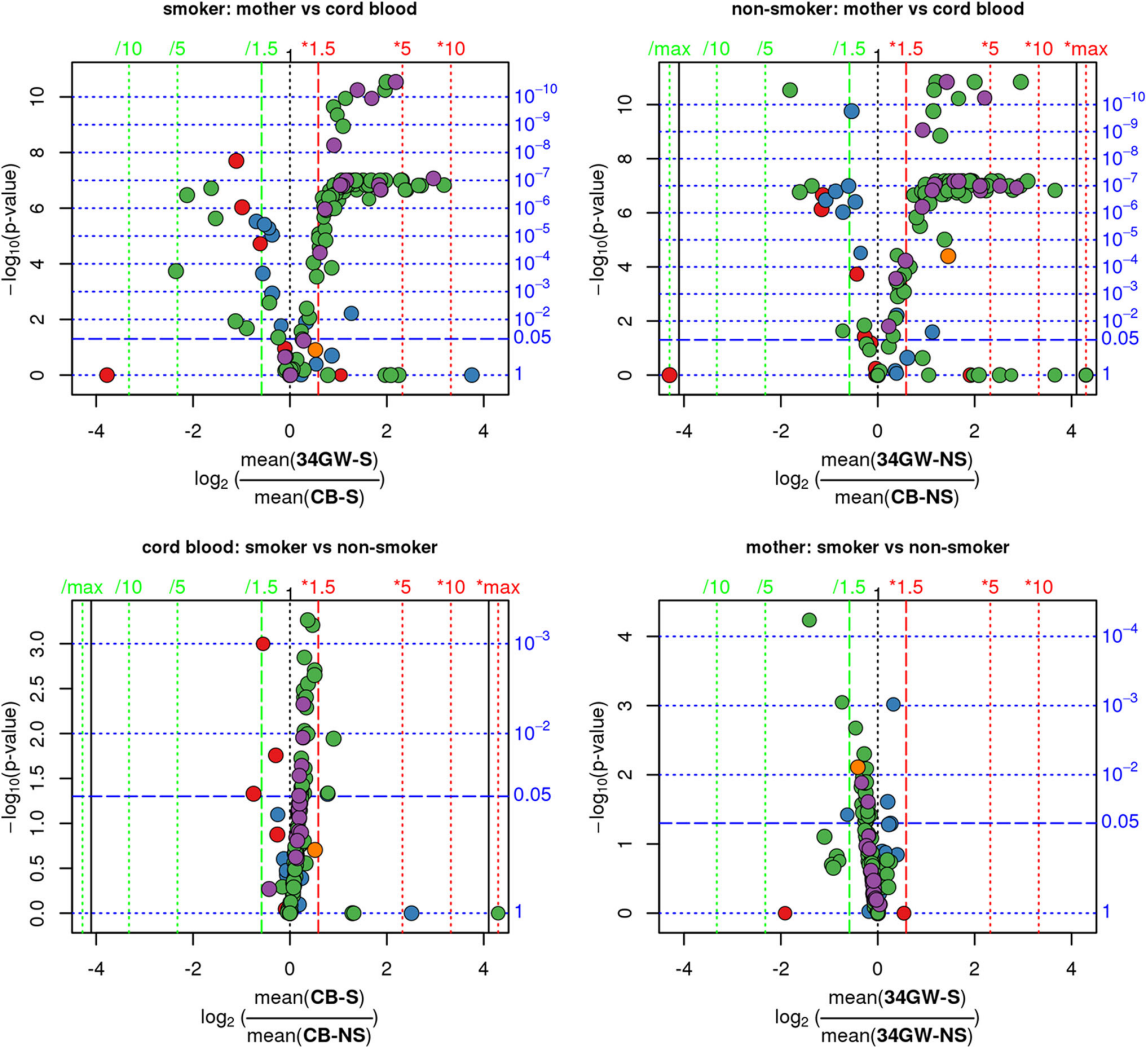
Volcano Plots of the metabolome profiles of mothers and children for smoker and non-smoker

In order to obtain information on whether smoking of the mother affects their own and the foetal endogenous metabolism, serum samples from the mothers and cord blood samples were analysed using a targeted metabolomics approach. This approach allows assessing 163 parameters from the substance classes of amino acids, acylcarnitines, hexoses, phosphatidylcholines and sphingomyelines. Based on PCA, the main cause for the variance (45 %) is based on the difference between mother and cord blood while there is no clear differentiation for all metabolites between the smoking and non-smoking groups. The stark differences between mother and cord blood are indicated by very low p-values for both the smoking and non-smoker samples (Fig. 2a, b). In both sample sets the maternal serum contains for example more Orn, SMC 24:0 and short chain PCaa/PCae, whereas the cord blood showed increased levels of Gln, Met and C18:2.

Beside the general differences the direction of the smoking-related changes is opposite in the two sample sets, mostly negative in children and mostly positive in the mothers. These results were validated by multiple measurements. Although this mirrored appearance is interesting we will focus in this study on the effects of smoking on the endogenous metabolome as such.

**Fig. 3:**
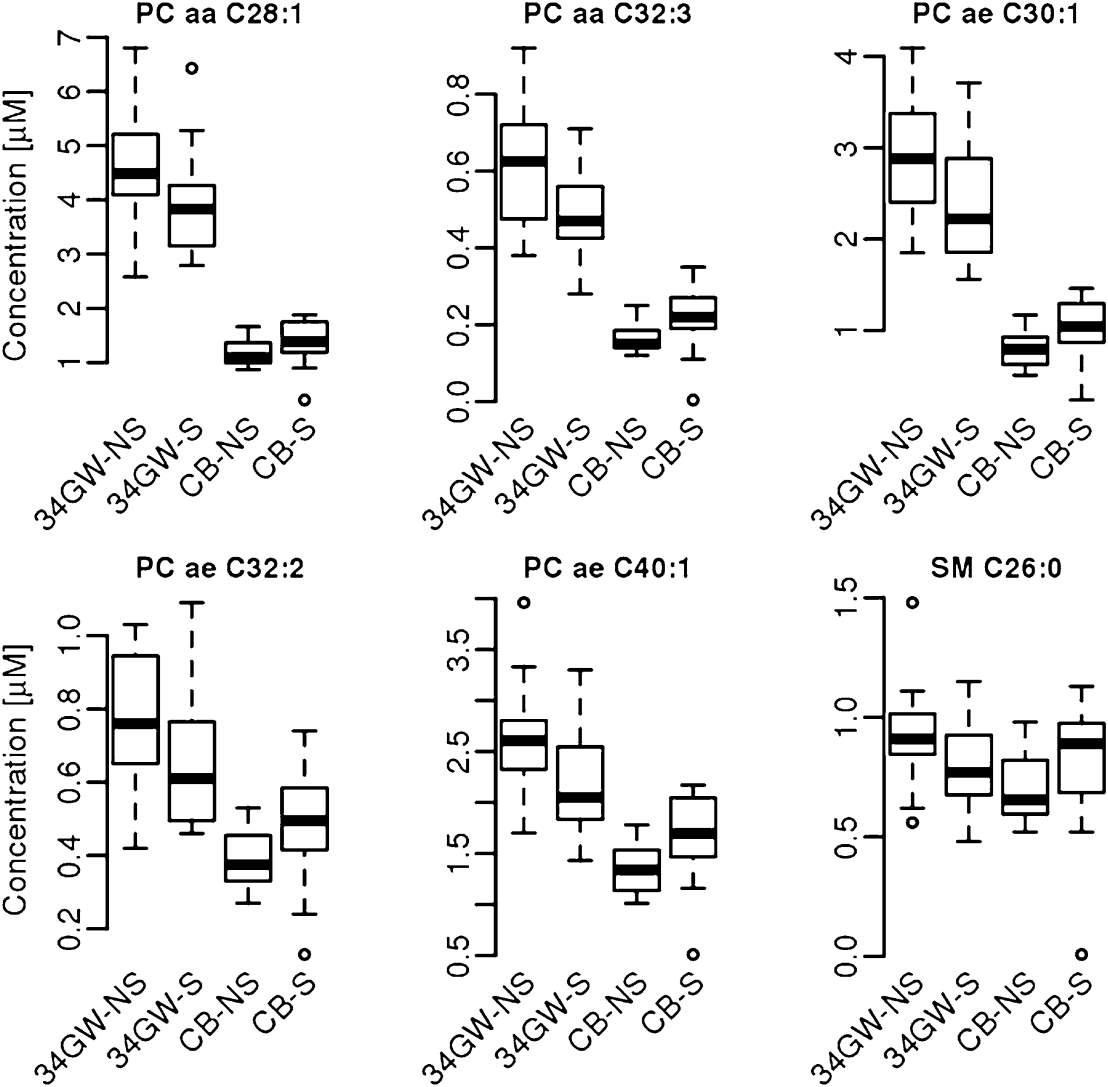
Smoke-dependent (*S* smoker, *NS* non-smoker) significantly affected metabolites of mothers at 34th week of gestation (34GW) and cord blood (CB)

Significant differences caused by smoking were found in different substance classes. Both the diacyl- as well as the acyl-ether phospholipids was determined as the most strongly affected substance classes, followed by the acyl carnitines and amino acids. More specifically, 23 significantly different abundant endogenous metabolites were found for the mothers and 25 for the foetus group in dependency to the smoking habit of the mother. For the 6 metabolites that were affected in both of the groups (PCaaC28:1, PCaaC32:3, PCaeC30:1, PCaeC32:2, PCaeC40:1, and SM C 26:0) detailed information is provided in the supplemental tables S1 and S2 and in Fig. 3. In the maternal samples smoking is related with an up regulation of amino acids and a down regulation of PCaa and PCae and SM molecules. In the foetal samples smoking increases all parameters, namely PCaa, PCae and SM, with the exception of acylcarnitines which were significantly down regulated.

**Fig. 4:**
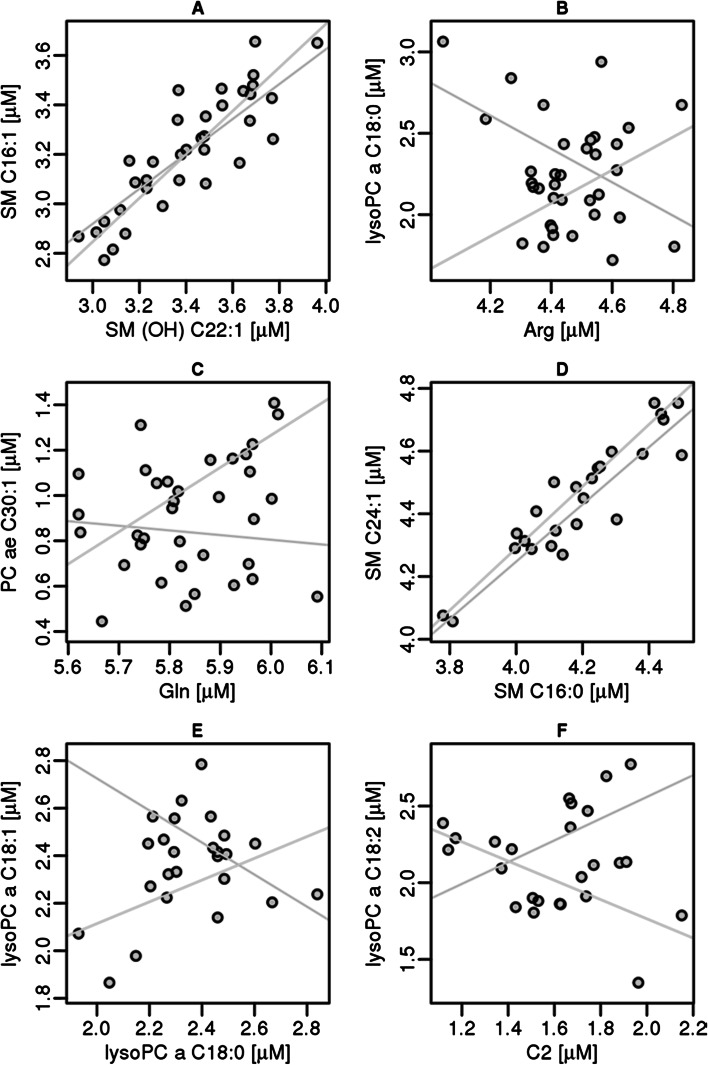
Correlations of metabolites between smokers (*blue*) and non-smokers (*green*) for mother (**a**, **b** and **c**) and cord blood (**d**, **e** and **f**) (Color figure online)

 In the next step of evaluation the correlation among the different metabolites within the groups of smoking mothers (supplemental Figure S1) and cord blood samples of the tobacco smoke exposed children (supplemental Figure S2) was analysed (see also Fig. 4). In the samples from the mothers positive correlations were detected for several groups of metabolites, namely for amino acids (group 1, Arg, Gln, Gly, His, Met, Thre, Trp and Tyr) the phosphatidylcholines with residues ranging from 28 to 44 and various acyl- and alkyl combinations (group 2), the set of lysophosphatidylcholines (group3) and the sphingomyelines (group 4). In the cord blood samples nearly the same groups were detected but the group of phosphatidylcholines was less well separated and also the group of lysophosphatidylcholines showed weaker correlations. For the group of amino acids the correlation nearly vanished. In addition to the groups found in the adults one additional group of positively correlating analytes, the carnitines were detected (Figure S2, group 5, C14:1, C15, C15:1, C18:1, C18:2, C2, C3, C3-DC/C4-OH, and C4).

In addition to the regular correlation approach, we inferred a Gaussian graphical model (GGM) from our metabolomics dataset. GGMs are based on *partial* correlation coefficients, which inherently eliminate *indirect* effects in the dataset and have been shown to recover metabolic pathways from plasma metabolomics data recently (Krumsiek et al. 2011a). The GGM was calculated on the combined dataset including all conditions, but corrected for confounding effects of mother and cord blood samples as well as smoking. The resulting network can be regarded as an interaction network capturing intrinsic metabolic relationships irrespective of the investigated phenotypes. An edge was included in the model if the respective partial correlation was significantly different from zero with α = 0.01 after FDR correction. The final network contained 123 nodes (one for each metabolite) and 91 out of 7503 theoretically possible edge (1.2 % connectivity).

In the following, we describe three selected subnetworks and smoking-related changes in both mothers and children (see supplementary material Figure S3). The first subnetwork comprises the five amino acids glutamine, tyrosine, methionine, phenylalanine and glycine and their reconstructed biochemical interactions (Figure S3-A). We observe a moderate, but coordinate up-regulation of these five amino acids upon smoking in mothers, whereas the same metabolites show lower concentrations in cord blood. An opposite effect can be seen for the phospholipid network shown in Figure S3-B. In this case, we observe a slight decrease upon smoking in mothers and an increase in cord blood. Similarly, the phospholipid network in Figure S3-C provides another evidence for a coordinate down-regulation of phospholipids in mothers and an up-regulation in cord blood for the smoking group. Interestingly, the GGM predicts an interaction between PCaaC32:3 and propionylcarnitine (C3) as well as free carnitine (C0), which show little to no effects with respect to smoking.

## Discussion

### Comparison to other nicotine dependent endogenous biomarkers

It is well known that cigarette smoke or passive smoking can have negative health effects. Around 4700 chemicals could be found in cigarette smoke (Repine, The Oxidative Stress Study 1997). The markers of SPMA and SBMA failed for separating the groups judged by statistics. In respect to SPMA, which is more often used due to the higher concentration of benzene in tobacco, smoke, this is in accordance to Barbieri et al. (2008) where also no correlation of the smoking habit and the SPMA levels were detected. But other studies have shown a correlation (Lovreglio et al. 2013) (Protano et al. 2010) (Lv et al. 2014). One possible explanation of this contradiction might be the different levels of exposure in those studies. In contrast cotinine is a reliable marker for smoking and the correlation has been established in many studies Benowitz et al. (2009) (Mattsson et al. 2015) (Martinez-Sanchez et al. 2014) The degree of correlation with the self-report questionnaires is in agreement with a recent study (Park et al. 2015) comparing questionnaires and urine sampling showing a tendency of underreporting in self-reports.

One potential mechanism for various adverse health effects can be smoke associated oxidative stress by influencing the antioxidant defence system (Alberg 2002). We confirm here the specific effects on the lipophilic part of the antioxidative stress response, which is in agree ment/disagreement with (Titova et al. 2012) and (Ardalic et al. 2014).

It was also shown that exposure to nicotine can trigger a wide range of metabolic changes (Wang-Sattler et al. 2008b). In an evaluation of metabolic profiles of smokers, former smokers and non-smokers, all men, moreover 23 lipid metabolites were identified as nicotine dependent biomarkers (Wang-Sattler et al. 2008a). In our study, including only pregnant women and newborn children, other smoking-specific metabolic profiles were found. The accordance to this study in terms of the exact molecules is limited, since only one metabolite, PCae40:6 was detected in both studies as a nicotine-dependent down regulated biomarker. Beyond PCae40.6 many similar molecules were identified as tobacco smoke related biomarkers, as for example PCaa34:3 in our study and the molecules PCaa34:1and PCaa34:2 by Wang-Sattler et al. More related molecules were found in the case of PCaa36:5 which was differential abundant in this study and PCaa36:2, PCaa36:1 and PCaa36:3 which were found by Wang-Sattler et al. In general, we detected more markers from the acyl-ether group of PC in our female exposure group, whereas in men more acyl–acyl forms were identified as tobacco smoke sensitive (Wang-Sattler et al.). Thereby, the direction of changes in respect to the PCaa molecules was highly similar. In both studies all significantly affected PCaa molecules were found to be upregulated. With respect to the PCae molecules Wang-Sattler et al. found only down-regulation while we observed mostly up-regulation in females.

The differences in these two studies might be due to the different source of samples, first the data set of Wang-Sattler et al. was larger especially for the non-smokers (255 not actually smoking people vs 28 actual smokers) and only male participants were included, whereas we analysed solely pregnant women and new-borns. A difference between the metabolism of male and female adult persons in respect to smoking was shown for total cholesterol, HDL-C and LDL-C in a recent study (Al-Bazi et al. 2011).

A higher sensitivity of female subjects towards tobacco smoke in terms of lipid metabolism would explain the differences between this study and the results from Wang-Sattler et al. In addition, 163 instead of 198 metabolites were assessed and some of the detected biomarkers were not included in our measurement, which might have also influenced the ranking of the biomarkers. We have also tested the gender dependent differences within the group of newborns but did not detect any significant differences between male and female samples. Consequently the gender of the newborns is also not responsible for the large difference between the mother and the newborns.

The differentially abundant metabolites fall predominantly into the group of lipids. And in general the concentrations of lipids are lowered in the cord blood. This leads to the hypothesis that the newborns use beside other nutrients like glucose and amino acids also the normal spectrum of serum lipids as nutrients.

### Correlations within and between groups of molecules

The correlation patterns of groups of metabolites underline the biochemical relations between them. As metabolites either originate from identical precursors or are transformed by the same enzymes or metabolic pathways they consequently often correlate in their abundance. Remarkably, the correlation patterns in the maternal and the foetal samples differed, most strongly in the group of carnitines, which were more strongly correlated in the foetal samples. The degree of correlation can be also taken as a hint for regulatory pressure on a group of molecules, since without it; the abundances can be expected to scatter more widely.

Acyl-carnitines are used in new-born screening for detecting fatty and organic acid metabolism disorders since 1998 (Marsden et al. 2006). In the foetus the metabolism is strongly driven towards fat oxidation (Melichar et al. 1965), in which acyl carnitines are crucially involved by the transport of acyl-CoA into the mitochondria. Thereby a reduced level of acyl carnitines might lead to a reduced energy production. The lowering effect of smoking on carnitine levels in the foetus might be one link in the mechanistic chain leading to lower birth weights in new-borns of smoking mothers.

Beside the expected correlation between the PCaa, PCae and SM groups the correlation between lysophosphatidylcholines on the one hand and the amino acids Gln, Gly, His and Met on the other hand was surprising. The link between lysophosphatidylcholines and glutamine was reported to be caused by the simultaneous induction of glutamine synthase and the increase of glycerol incorporation into arachidonic acid as a result of stimulating glioma cells (Sun et al. 1997). Glutamine has also been reported to be involved in glucose and glycogen metabolism, immune system function and the maintenance of skeletal muscle (Antonio and Street 1999). Glutamine is linked with histidine synthesis via glutamate, which was not determined in this assay, so this linkage remains hypothetical.

Glycine is involved in many different biochemical mechanisms including the regulation of plasma cholesterol and triglyceride levels (Ratnayake et al. 1997), but the specific linkage to lysophosphatidylcholines and not to other molecules from the fatty acid metabolism will require further investigation.

### Origin of biomarkers in the endogenous metabolome and metabolomic programming

The first hindrance of a straight-forward interpretation of serum metabolome studies lies in the uncertain origin of the metabolites. Only a minority of metabolites is reported to be produced in the serum itself, instead most stem from liver, skeletal muscle, fat tissue and brain (Suhre et al. 2010). Some of the metabolites like glycine and glutamine are known to be actively released into the blood with a reported function (Ratnayake et al. 1997). Others like carnitines seem to leak out of for example muscle cells with a decreased mitochondrial activity (data not shown). The PCaa and PCae as well as SM molecules are supposed to be part of the cell membrane and so their leakage into the blood stream can be a direct consequence of membrane damage caused by substances in ETS especially of blood cells, where it is also reported to alter the lipid composition (Gangopadhyay et al. 2012; Padmavathi et al. 2010). Then one would expect that the profile of lipids resembles their occurrence of in the membrane. This assumption would also demand for only up-regulation of lipid-related molecules and not for an ETS specific down-regulation as it was found in this study and earlier for specific lipids also by Wang-Sattler et al. (2008b). Furthermore, in studies on the serum metabolome of diabetes patients also changes of PCaa and PCae were detected (Suhre et al. 2010) and correlated with the known disturbed lipid homeostasis in diabetic patients. The underlying details like which organ is secreting which lipid by which mechanism will be an important research question for the future.

The mechanistic link from an altered metabolism early in life towards health effects later in life is given by metabolomic programming, describing the hypothesis that e.g. nutritional impact early in life has long reaching consequences (Godfrey and Barker 2000). Beside nutrition as an exogenous factor also tobacco smoke exposure may affect the metabolome with consequences for the disease risk. In the case of nicotine this has been proven since in mice the maternal nicotine exposure resulted in increased postnatal body weight and higher levels of body fat in the foetus (Gao et al. 2005; Somm et al. 2008). This is in contrast to the findings in humans, where smoking generally leads to a reduced body weight of new-borns of smoking mothers. But this effect is reported to be overcompensated by increased rates of weight gain, which seem to result in an increased risk for metabolic disorders (Harrod et al. 2015).

These effects of smoking direct the attention towards effects on the central endocrine control of body weight homeostasis, which might be explained by nicotine dependent alterations in expression of neuropeptide Y and propiomelanocortin (Grove et al. 2001). Also in toxicological studies on foetal rats an influence of maternal nicotine exposure has led to alterations in the hypothalamus–pituitary–adrenal axis with consequences on expression levels of 11ß-hydroxysteroid dehydrogenase-2, glucocorticoid receptor and insulin-like growth factor, the corresponding IGF-1 receptor and insulin receptor in the foetal liver (Xu et al. 2012). Following answers of our hypotheses are possible. Mothers and children show different regulation of metabolom. That is also affected by the smoking behaviour of mothers. Urine cotinine measurements are useful to describe the smoke behaviour of mothers. It could be shown, that the antioxidative status of lipid soluble fraction is is negative influenced by smoking.

### Known limitations of this study

Serum samples from mothers and babies were collected at different time points. This may lead to inconsistencies, because metabolite concentrations may differ between 34th week of pregnancy (sample taken from mother) and day of birth (sample taken from cord blood). Due to ethical reasons we don’t asked the mothers for a blood sample during birth process. Despite of this limitation, we assume a steady state of smoking related metabolites during this time frame.

## Conclusion

With this study we demonstrate that serum from cord blood has become a suitable medium for metabolomic profiling that provides valuable information on the foetal metabolism. Furthermore, we describe effects of ETS on the foetal metabolome which is affected in a different way than the maternal metabolome, pointing also to different underlying mechanisms in adults and newborn children.

## Electronic supplementary material

Supplementary material 1 (PPTX 3824 kb)

## Abbreviations

ACL: Antioxidative capacity of lipid soluble compounds
ACW: Antioxidative capacity of water soluble compounds
CB: Cord blood
GW: Gestational week
S: Smoke burdened
lysoPC: Lysophosphatidylcholines
NS: No smoke burden
PC: Phosphatidylcholines (a, a-diacyl form; a, e acyl-ether form)
SM: Sphingomyelines
SBMA: S-Benzyl mercapturic acid
SPMA: S-Phenyl mercapturic acid
