# Supplementary material for: Metabolomics reveals effects of maternal smoking on endogenous metabolites from lipid metabolism in cord blood of newborns
Source: Metabolomics. 2016 Mar 8;12:76. doi: 10.1007/s11306-016-0983-z (PMC4783445; doi:10.1007/s11306-016-0983-z)
Supplement: Supplementary file 1 — Supplementary material 1 (PPTX 3824 kb) [file 11306_2016_983_MOESM1_ESM.pptx]

## Slide 1
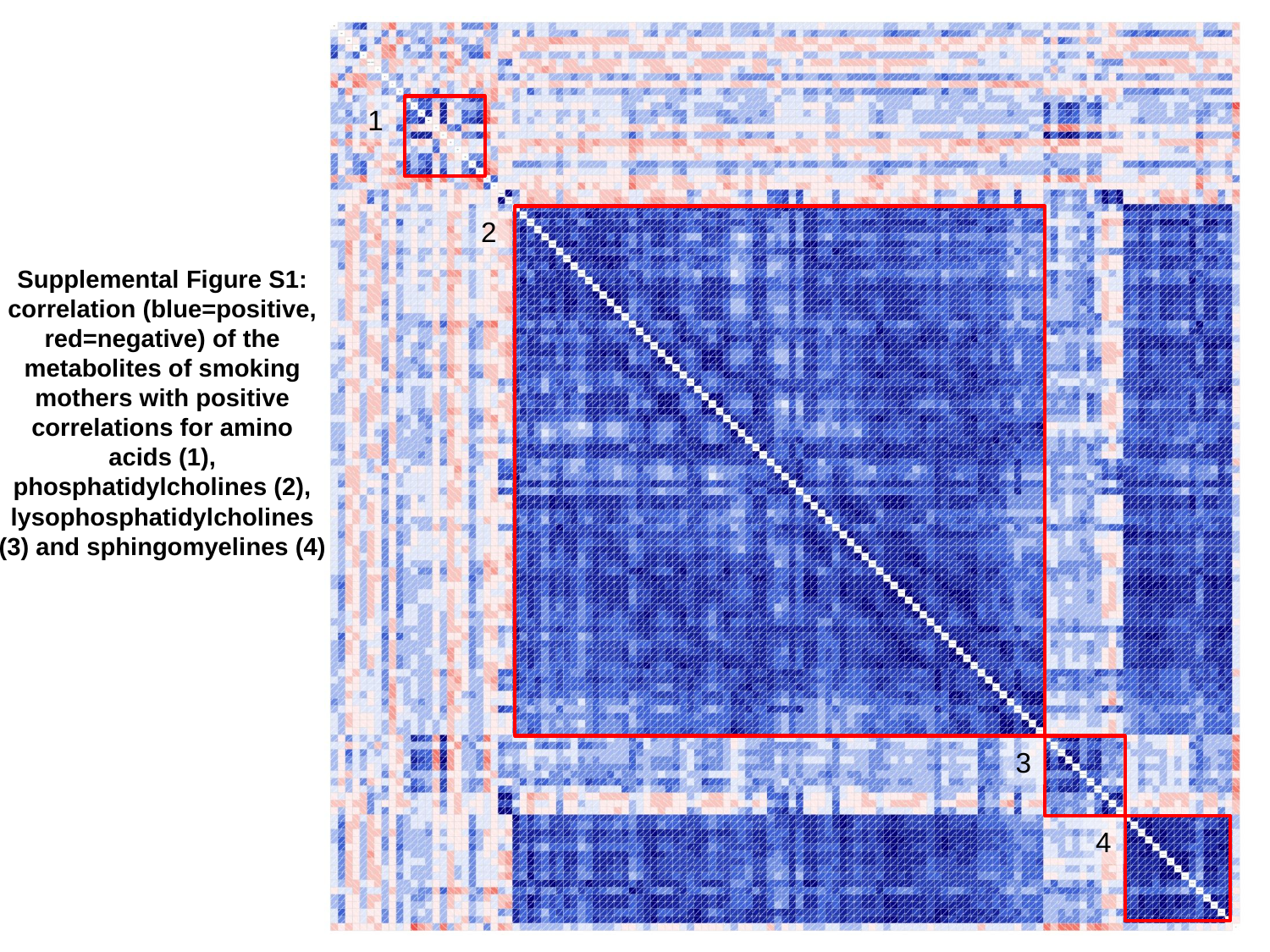

1
2
# Supplemental Figure S1: correlation (blue=positive, red=negative) of the metabolites of smoking mothers with positive correlations for amino acids (1), phosphatidylcholines (2), lysophosphatidylcholines (3) and sphingomyelines (4)
3
4

## Slide 2
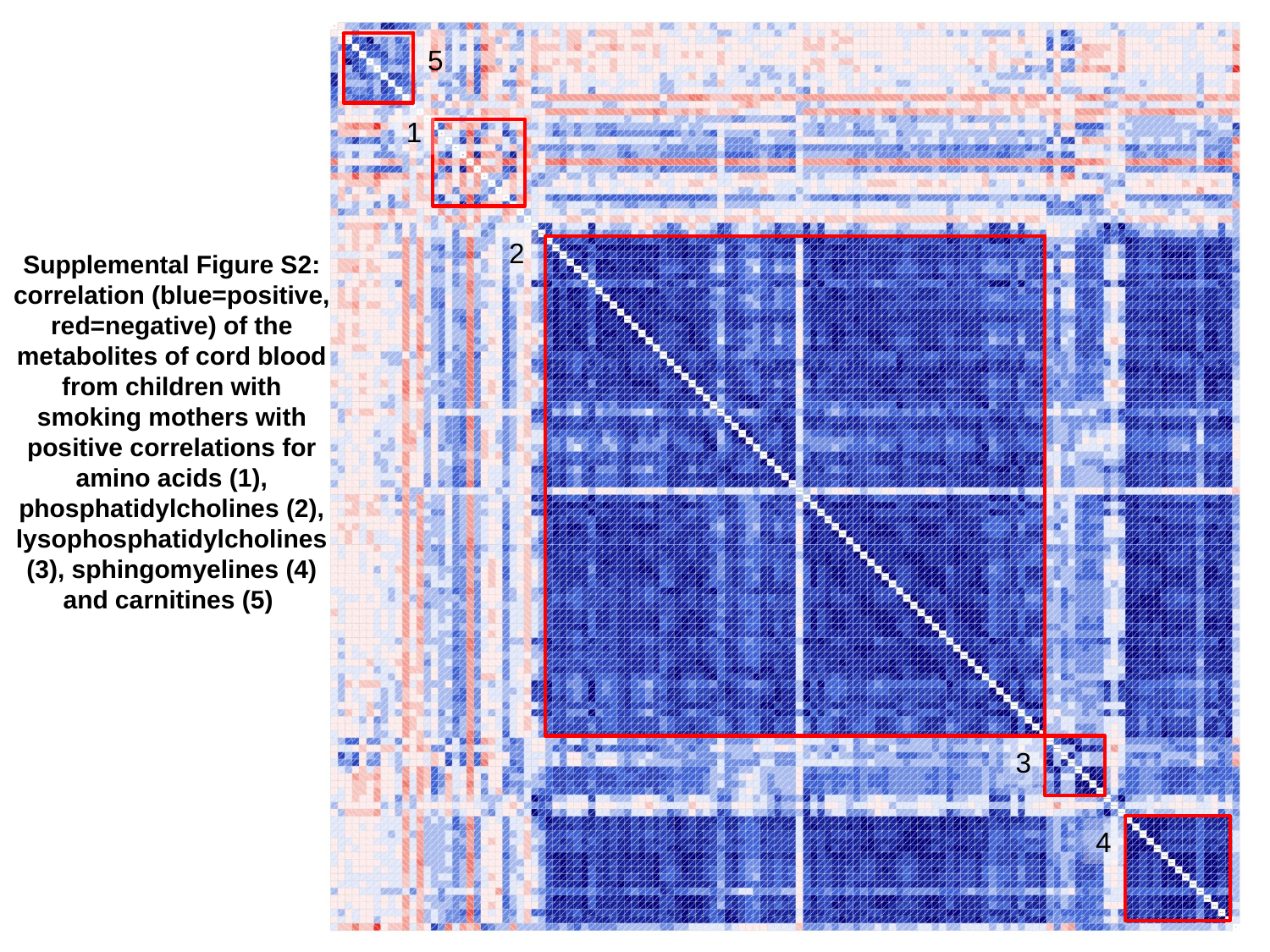

5
1
2
Supplemental Figure S2: correlation (blue=positive, red=negative) of the metabolites of cord blood from children with smoking mothers with positive correlations for amino acids (1), phosphatidylcholines (2), lysophosphatidylcholines (3), sphingomyelines (4) and carnitines (5)
3
4

## Slide 3
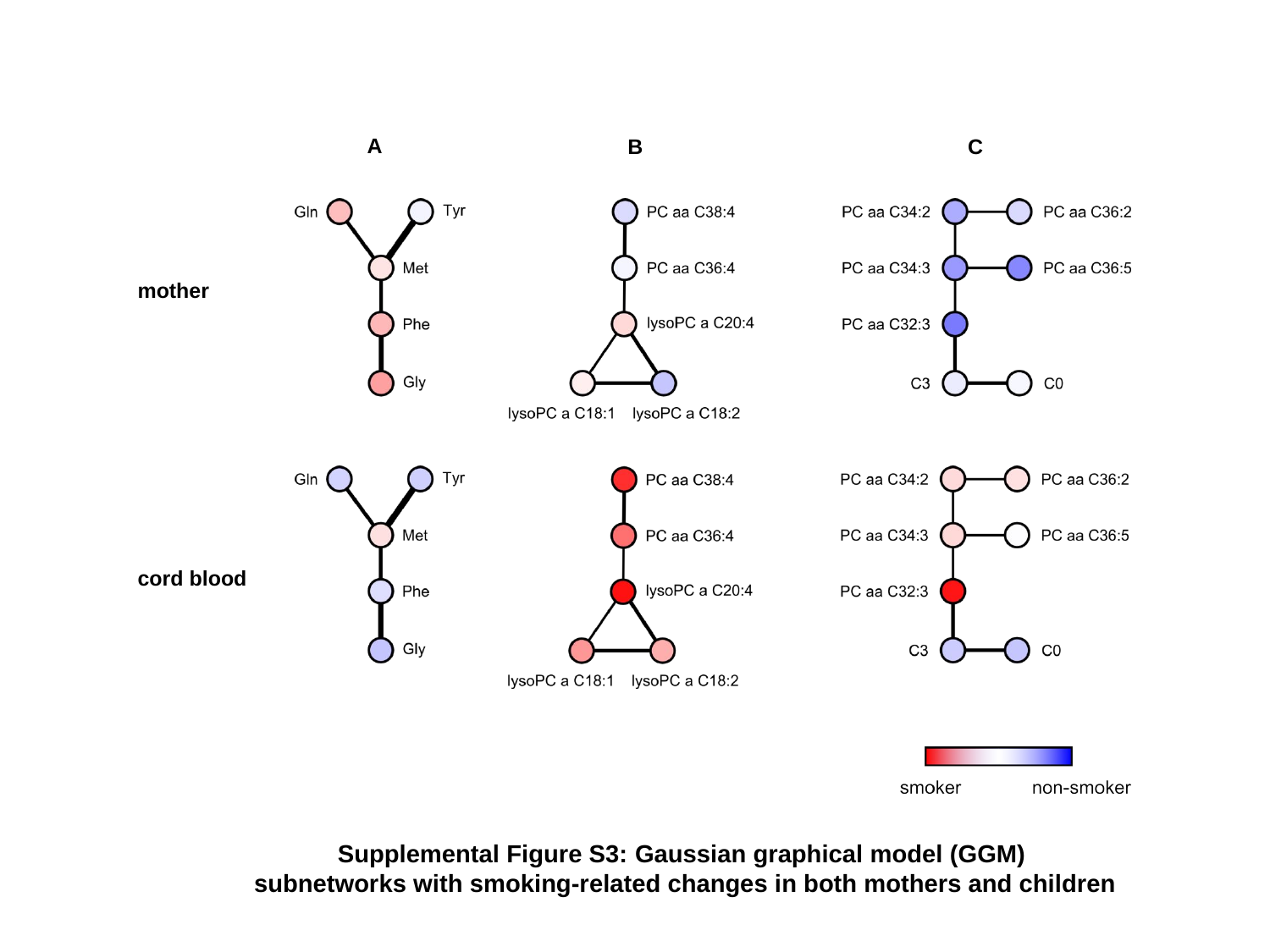

A
B
C
mother
cord blood
Supplemental Figure S3: Gaussian graphical model (GGM)
subnetworks with smoking-related changes in both mothers and children

## Slide 4
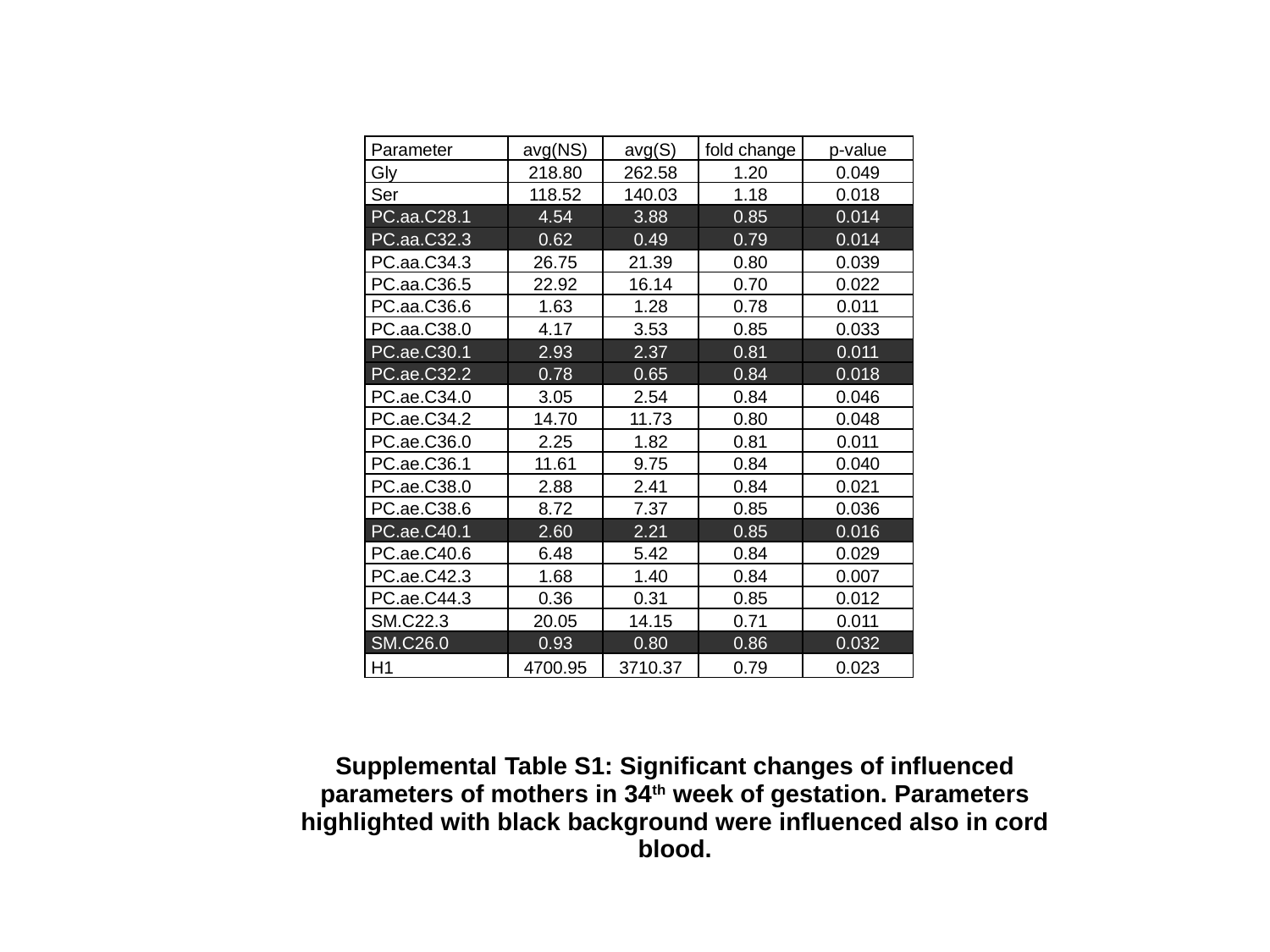

| Parameter | avg(NS) | avg(S) | fold change | p-value |
| --- | --- | --- | --- | --- |
| Gly | 218.80 | 262.58 | 1.20 | 0.049 |
| Ser | 118.52 | 140.03 | 1.18 | 0.018 |
| PC.aa.C28.1 | 4.54 | 3.88 | 0.85 | 0.014 |
| PC.aa.C32.3 | 0.62 | 0.49 | 0.79 | 0.014 |
| PC.aa.C34.3 | 26.75 | 21.39 | 0.80 | 0.039 |
| PC.aa.C36.5 | 22.92 | 16.14 | 0.70 | 0.022 |
| PC.aa.C36.6 | 1.63 | 1.28 | 0.78 | 0.011 |
| PC.aa.C38.0 | 4.17 | 3.53 | 0.85 | 0.033 |
| PC.ae.C30.1 | 2.93 | 2.37 | 0.81 | 0.011 |
| PC.ae.C32.2 | 0.78 | 0.65 | 0.84 | 0.018 |
| PC.ae.C34.0 | 3.05 | 2.54 | 0.84 | 0.046 |
| PC.ae.C34.2 | 14.70 | 11.73 | 0.80 | 0.048 |
| PC.ae.C36.0 | 2.25 | 1.82 | 0.81 | 0.011 |
| PC.ae.C36.1 | 11.61 | 9.75 | 0.84 | 0.040 |
| PC.ae.C38.0 | 2.88 | 2.41 | 0.84 | 0.021 |
| PC.ae.C38.6 | 8.72 | 7.37 | 0.85 | 0.036 |
| PC.ae.C40.1 | 2.60 | 2.21 | 0.85 | 0.016 |
| PC.ae.C40.6 | 6.48 | 5.42 | 0.84 | 0.029 |
| PC.ae.C42.3 | 1.68 | 1.40 | 0.84 | 0.007 |
| PC.ae.C44.3 | 0.36 | 0.31 | 0.85 | 0.012 |
| SM.C22.3 | 20.05 | 14.15 | 0.71 | 0.011 |
| SM.C26.0 | 0.93 | 0.80 | 0.86 | 0.032 |
| H1 | 4700.95 | 3710.37 | 0.79 | 0.023 |
# Supplemental Table S1: Significant changes of influenced parameters of mothers in 34th week of gestation. Parameters highlighted with black background were influenced also in cord blood.

## Slide 5
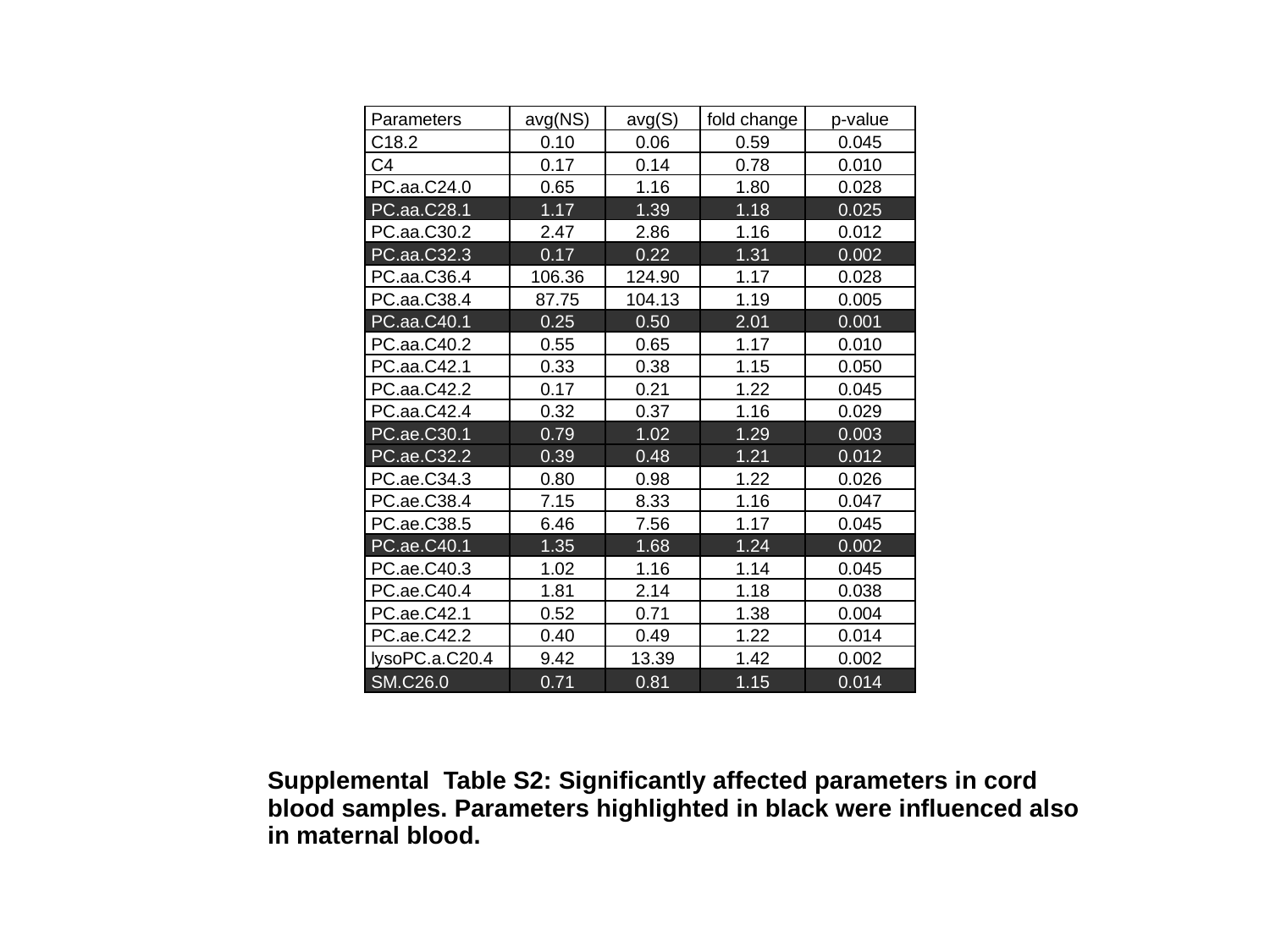

| Parameters | avg(NS) | avg(S) | fold change | p-value |
| --- | --- | --- | --- | --- |
| C18.2 | 0.10 | 0.06 | 0.59 | 0.045 |
| C4 | 0.17 | 0.14 | 0.78 | 0.010 |
| PC.aa.C24.0 | 0.65 | 1.16 | 1.80 | 0.028 |
| PC.aa.C28.1 | 1.17 | 1.39 | 1.18 | 0.025 |
| PC.aa.C30.2 | 2.47 | 2.86 | 1.16 | 0.012 |
| PC.aa.C32.3 | 0.17 | 0.22 | 1.31 | 0.002 |
| PC.aa.C36.4 | 106.36 | 124.90 | 1.17 | 0.028 |
| PC.aa.C38.4 | 87.75 | 104.13 | 1.19 | 0.005 |
| PC.aa.C40.1 | 0.25 | 0.50 | 2.01 | 0.001 |
| PC.aa.C40.2 | 0.55 | 0.65 | 1.17 | 0.010 |
| PC.aa.C42.1 | 0.33 | 0.38 | 1.15 | 0.050 |
| PC.aa.C42.2 | 0.17 | 0.21 | 1.22 | 0.045 |
| PC.aa.C42.4 | 0.32 | 0.37 | 1.16 | 0.029 |
| PC.ae.C30.1 | 0.79 | 1.02 | 1.29 | 0.003 |
| PC.ae.C32.2 | 0.39 | 0.48 | 1.21 | 0.012 |
| PC.ae.C34.3 | 0.80 | 0.98 | 1.22 | 0.026 |
| PC.ae.C38.4 | 7.15 | 8.33 | 1.16 | 0.047 |
| PC.ae.C38.5 | 6.46 | 7.56 | 1.17 | 0.045 |
| PC.ae.C40.1 | 1.35 | 1.68 | 1.24 | 0.002 |
| PC.ae.C40.3 | 1.02 | 1.16 | 1.14 | 0.045 |
| PC.ae.C40.4 | 1.81 | 2.14 | 1.18 | 0.038 |
| PC.ae.C42.1 | 0.52 | 0.71 | 1.38 | 0.004 |
| PC.ae.C42.2 | 0.40 | 0.49 | 1.22 | 0.014 |
| lysoPC.a.C20.4 | 9.42 | 13.39 | 1.42 | 0.002 |
| SM.C26.0 | 0.71 | 0.81 | 1.15 | 0.014 |
Supplemental Table S2: Significantly affected parameters in cord blood samples. Parameters highlighted in black were influenced also in maternal blood.
